# IGF-IR Internalizes with Caveolin-1 and PTRF/Cavin in Hacat Cells

**DOI:** 10.1371/journal.pone.0014157

**Published:** 2010-11-30

**Authors:** Barbara Salani, Mario Passalacqua, Sara Maffioli, Lucia Briatore, Meriem Hamoudane, Paola Contini, Renzo Cordera, Davide Maggi

**Affiliations:** 1 Department of Endocrinology and Medicine (DiSEM), University of Genova, Genova, Italy; 2 Department of Experimental Medicine (DIMES), Centre of Excellence for Biomedical Research, University of Genova, Genova, Italy; 3 Italian Institute of Biostructures and Biosystems (INBB), University of Genova, Genova, Italy; 4 Department of Internal Medicine and Medical Specialties (DIMI), University of Genova, Genova, Italy; Universidade Federal do Rio de Janeiro, Brazil

## Abstract

**Background:**

Insulin-like growth factor-I receptor (IGF-IR) is a tyrosine kinase receptor (RTK) associated with caveolae, invaginations of the plasma membrane that regulate vesicular transport, endocytosis and intracellular signaling. IGF-IR internalization represents a key mechanism of down-modulation of receptors number on plasma membrane. IGF-IR interacts directly with Caveolin-1 (Cav-1), the most relevant protein of caveolae. Recently it has been demonstrated that the Polymerase I and Transcript Release Factor I (PTRF/Cavin) is required for caveolae biogenesis and function. The role of Cav-1 and PTRF/Cavin in IGF-IR internalization is still to be clarified.

**Methodology/Principal Findings:**

We have investigated the interaction of IGF-IR with Cav-1 and PTRF/Cavin in the presence of IGF1in human Hacat cells. We show that IGF-IR internalization triggers Cav-1 and PTRF/Cavin translocation from plasma membrane to cytosol and increases IGF-IR interaction with these proteins. In fact, Cav-1 and PTRF/Cavin co-immunoprecipitate with IGF-IR during receptor internalization. We found a different time course of co-immunoprecipitation between IGF-IR and Cav-1 compared to IGF-IR and PTRF/Cavin. Cav-1 and PTRF/Cavin silencing by siRNA differently affect surface IGF-IR levels following IGF1 treatment: Cav-1 and PTRF/Cavin silencing significantly affect IGF-IR rate of internalization, while PTRF/Cavin silencing also decreases IGF-IR plasma membrane recovery. Since Cav-1 phosphorylation could have a role in IGF-IR internalization, the mutant Cav-1Y14F lacking Tyr14 was transfected. Cav-1Y14F transfected cells showed a reduced internalization of IGF-IR compared with cells expressing wild type Cav-1. Receptor internalization was not impaired by Clathrin silencing. These findings support a critical role of caveolae in IGF-IR intracellular traveling.

**Conclusions/Significance:**

These data indicate that Caveolae play a role in IGF-IR internalization. Based on these findings, Cav-1 and PTRF/Cavin could represent two relevant and distinct targets to modulate IGF-IR function.

## Introduction

Insulin like growth factor I receptor (IGF-IR) is a tyrosine kinase receptor (RTK) that regulates cell proliferation and survival both in normal and malignant phenotypes [1]. IGF-IR plasma membrane compartmentalization could affect its downstream signaling and activation [2], [3]. Binding of IGF1 to the IGF-IR results in receptor auto phosphorylation, internalization and intracellular signaling pathway activation [4]. The mechanism by which RTKs number is regulated on cell surface is a balance between the rate of internalization and the rate of replacement (recycling and new synthesis). RTKs internalization is commonly triggered by ligand binding and occurs via clathrin-coated pits, the first identified and best studied route for entry of RTKs into the cell. Clathrin-coated pits take part to IGF-IR internalization [5], [6] but recently it has been demonstrated that different plasma membrane micro-domains such as caveolae could regulate the biological actions of many plasma membrane receptors [7], [8].

Caveolae are a subset of lipid rafts which regulate protein endocytosis and intracellular trafficking, cholesterol homeostasis, and signal transduction [9]. Cav-1 is the principal protein of caveolae [10].Caveolae are dependent on Cav-1 expression [11], [12]. Recently it has been demonstrated that the stability of caveolae could be affected also by Polymerase I Transcript Release Factor or Cavin (PTRF/Cavin), originally described as a nuclear protein [13]. PTRF/Cavin is a regulator of caveolae biogenesis and represents the first member of a family of proteins called PTRF/Cavin-related proteins identified as regulators of caveolae functions [14], [15]. PTRF/Cavin co-immunoprecipitates with Cav-1 [16], and its silencing disrupts caveolae organization [15]. Moreover, PTRF/Cavin could participate actively to signaling processes that start from cell surface, as demonstrated by PTRF/Cavin translocation from plasma membrane to the nucleus in presence of Insulin [17].

Caveolae are involved in IGF-IR downstream signaling. In fact, IGF-IR and its substrates are present and activated in caveolae [18], [19]. IGF-IR interacts directly with Cav-1 [2]. Several experimental findings suggest that IGF-IR signaling could be regulated by Cav-1. Cav-1 is tyrosine phosphorylated (PY14) upon IGF1 stimulation and redistributes on plasma membrane patches [20], [21]. It remains to be establish whether caveolae could act as inhibitors or activators of IGF-IR signaling [8]. In Cav-1 silenced cells, activation of IGF-IR as well as phosphorylation of its proximal downstream substrates IRS-1 and Shc are greatly reduced. Down regulation of Cav-1 causes also a decreased activation of Akt kinase that participates to the anti-apoptotic function of IGF-IR [2].

While it has been demonstrated the involvement of caveolae during endocytic processes, it is yet to be clarified whether Cav-1 and PTRF/Cavin could play a role to regulate IGF-IR surface levels following IGF1 treatment.

Here we demonstrate that: 1) IGF1 increases the co-IP of PTRF/Cavin and Cav-1 with IGF-IR; 2) Cav-1, PTRF/Cavin and IGF-IR co-localize on plasma membrane and IGF1causes their internalization; 3) Cav-1 and PTRF/Cavin silencing decreases IGF-IR internalization; 4) PTRF/Cavin silencing affects IGF-IR rate of replacement on cell surface. 5) Phosphorylation of Cav-1 protein at tyrosine 14 plays a role to sustain IGF-IR traveling to the cytoplasm.

## Results

IGF-IR localization in caveolae has been consistently demonstrated [8], but the role of caveolae in IGF-IR internalization is still unknown. Since Cav-1 and PTRF/Cavin are important components of caveolae [15], [22], [23] we tested the hypothesis that Cav-1 and PTRF/Cavin could affect IGF-IR intracellular traveling in HaCaT keratinocytes, a cell line that constitutively expresses Cav-1 [24], PTRF/Cavin and IGF-IR.

**Figure 1:**
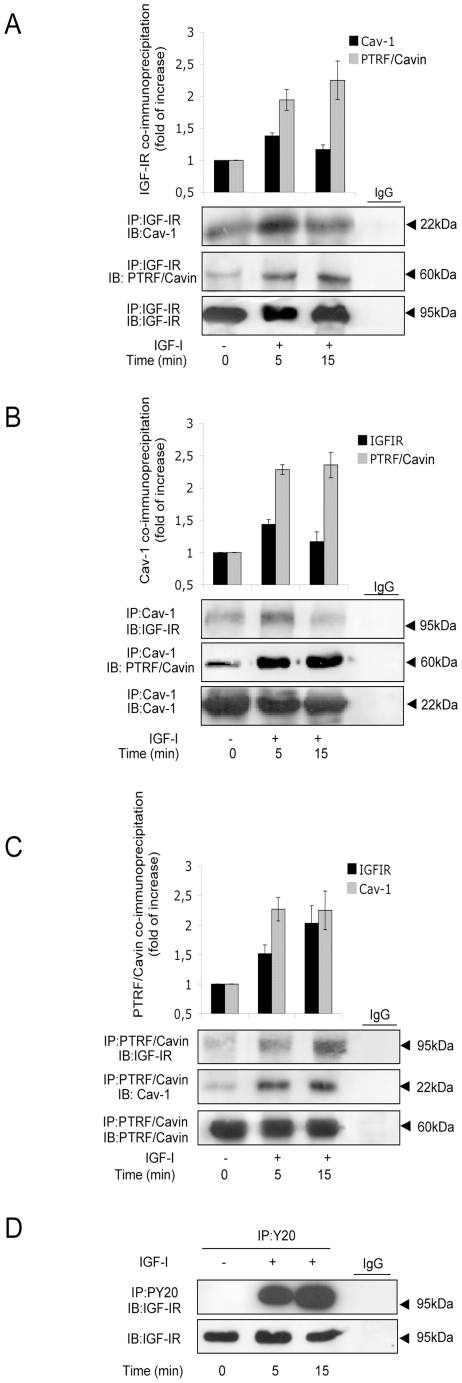
Cav-1 and PTRF/Cavin co-immunoprecipitation with IGF-IR. Serum starved HaCat cells were stimulated with IGF110 nM for the indicated times and then lysed. Equal amount of cell lysates were immunoprecipitated (IP) and immunoblotted (IB) with the indicated antibodies. The graphs represent quantification of co-immunoprecipitation experiments following densitometric analysis of bands and are expressed as fold of increase. Data shown are representative of three independent experiments and are expressed as the mean ± SD.

**Figure 2:**
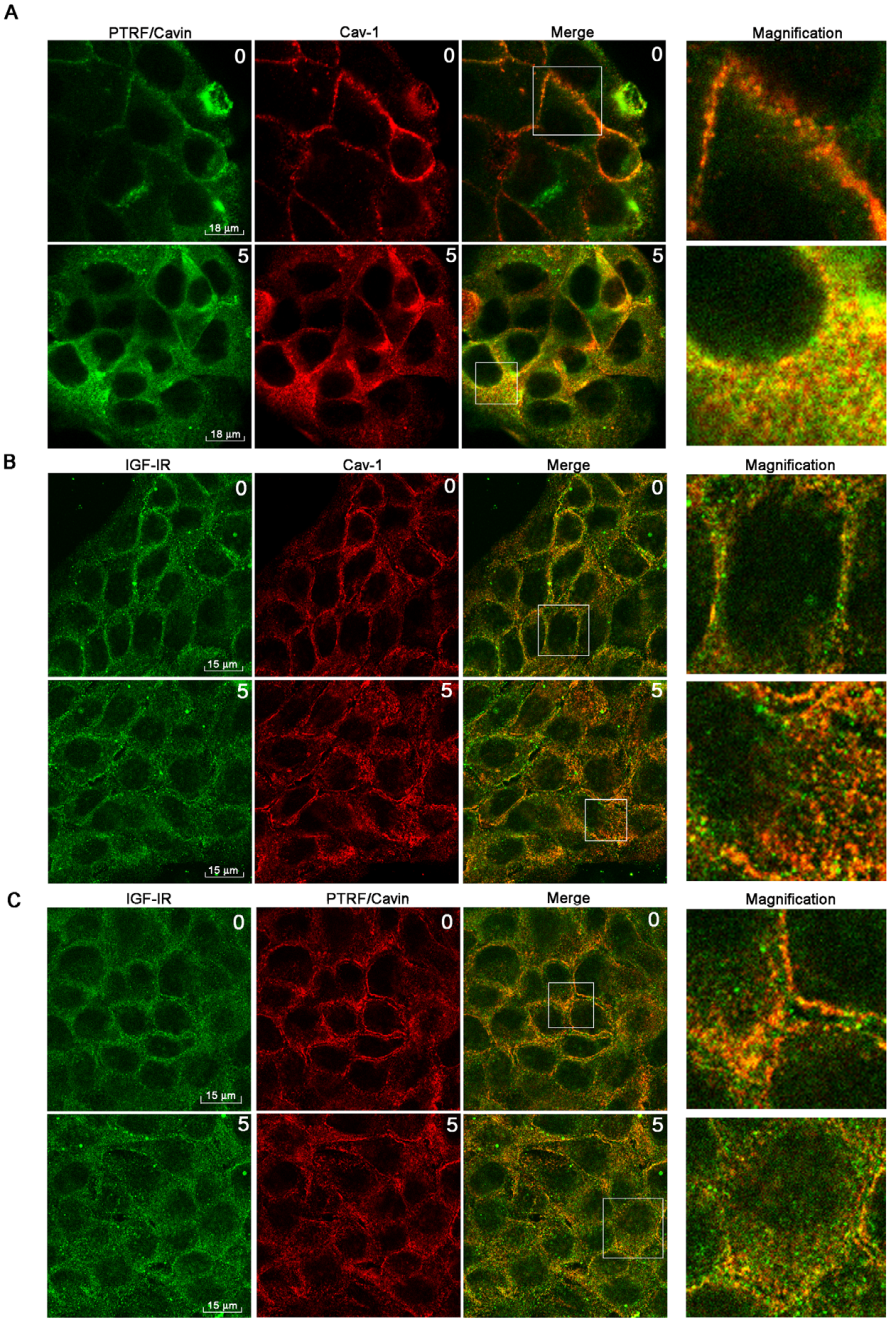
IGF1induces PTRF/Cavin and Cav-1 internalization. HaCat cells were treated with IGF1 (10 nM) for 5 min, fixed in 4% formaldehyde, permeabilized with Methanol at −20°C, labeled with (A) a rabbit anti-PTRF/Cavin (red) and a mouse anti-Cav-1 (green), or (B) a mouse anti-IGF-IR (green) and a rabbit anti-Cav-1 antibody (red), or alternatively (C) with a mouse anti-IGF-IR (green) and a rabbit anti-PTRF/Cavin (red) antibody and imaged by confocal immunofluorescence microscopy. Column 4 shows magnified fields indicated by squares in column 3. Merged fields show co-localization (yellow) respectively of PTRF/Cavin and Cav-1 (A), IGFIR and Cav-1 (B) and of IGF-IR and PTRF (C).

We investigated whether PTRF/Cavin, could physically interact with IGF-IR during IGF-IR activation. HaCat cells were stimulated with IGF1 10 nM and lysed. We performed reciprocal co-immunoprecipitations between IGF-IR, Cav-1 and PTRF. The time course of IGF-IR and Cav-1 co-immunoprecipitation showed a maximum at 5 min (Fig. 1A,1B), while PTRF/Cavin and IGF-IR co-immunoprecipitation increased till 15 min (Fig. 1A,1C) and was associated with IGF-IR tyrosine phosphorylation (Fig. 1D). The graphs represent quantification of co-immunoprecipitation experiments following densitometric analysis of bands. Sub-cellular localization of IGF-IR with PTRF/Cavin and Cav-1 was studied by confocal microscopy. Consistently with previous reports [23] in basal condition both Cav-1 and PTRF/Cavin co-localized in plasma membrane showing a distribution typical of caveolae staining-pattern (Fig.2A). IGF1 induced Cav-1 and PTRF/Cavin internalization as shown by the punctuate staining of the two proteins in the cytoplasm (Fig.2A). IGF-IR showed a cell surface staining which co-localized with Cav-1 (Fig.2B) and PTRF/Cavin (Fig.2C). IGF1induced cytoplasmatic co-localization of IGF-IR with Cav-1 and PTRF/Cavin suggesting that IGF1treatment resulted in Cav-1 and PTRF/Cavin redistribution from plasma membrane to intracellular compartment. Quantification of co-localization between IGF-IR/Cav-1 and IGF-IR/PTRF did not reveal any significant change before and after 5′ min of IGF-1 treatment (IGF-IR/Cav-1: 71%±8 *vs.* 66%±11; IGF-IR/PTRF: 50%±11 *vs*. 51%±13).

**Figure 3:**
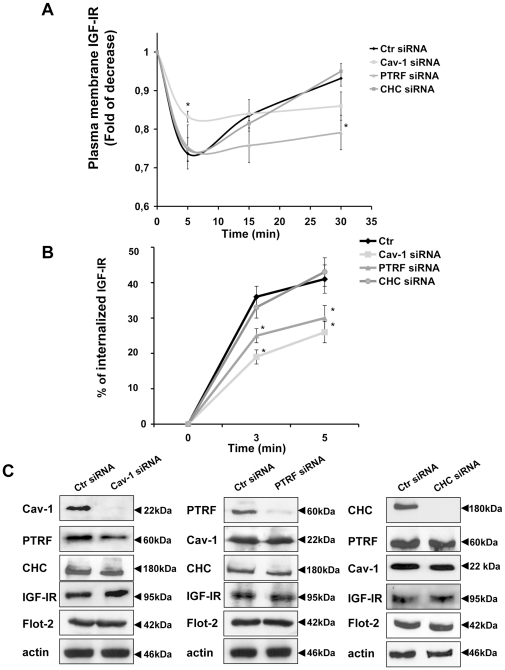
Cav-1 and PTRF/Cavin are required for IGF-IR internalization and plasma membrane recovery. HaCat cells were transfected with siRNA for Cav-1 (Cav-1-siRNA), for PTRF/Cavin (PTRF/Cavin-siRNA), for Clathrin Heavy Chain (Clathrin HC-siRNA) and with scrambled control siRNA (Ctr-siRNA) as described in materials and methods. (A) 72 hours after transfection, serum-starved cells were treated with IGF110 nM for the indicated times, trypsined, washed, blocked and incubated with a mouse PE-conjugated IGF-IR antibody. PE-conjugated IGF-IR labeled cells were analyzed by flow-cytometry to measure plasma membrane IGF-IR expression as described in Materials and Methods. (B) Ctr-siRNA, Cav-1-siRNA, PTRF/Cavin-siRNA and Clathrin HC-siRNA HaCat cells were serum-starved and subjected to a biotinylation based endocytic assay with NH-SS-biotin at 4°C (see Materials and Methods). The cells were then warmed at 37°C with medium containing IGF110 nM to allow IGF-IR internalization. Glutathione was used to reduce the proteins not internalized from the plasma membrane. IGF-IR was immunoprecipitated with IGF-IR antibody and the internalized IGF-IR was revealed by Western Blot with a Streptavidin-HRP antibody. Data were quantified using NIH-Image and plotted in the graph. The amount of biotinylated internalized IGF-IR was expressed as a percentage of the amount of IGF-IR on the surface at 4°C which we set as 100%. (C) 72 hours from the transfection serum-starved cells were lysed and equal amount of Ctr-siRNA and Cav-1-siRNA or Ctr-siRNA and PTRF/Cavin-siRNA and Clathrin HC-siRNA cell lysates were separated on SDS–PAGE, transferred on nitrocellulose and blotted with an antibody directed against Cav-1, PTRF/Cavin, Clathrin HC, IGF-IR, Flotillin-2 and actin proteins. Data are expressed as the mean ± SD. Statistical analysis was performed using Student's *t* test. *p<0.05.

Next, to determine the role of Cav-1 and PTRF/Cavin in IGF-IR internalization, we silenced Cav-1 and PTRF/Cavin by siRNA and verified by FACS analysis the effect on IGF-IR surface levels in presence of IGF-I. To exclude a role of Clathrin coated pits in IGF-IR intracellular traveling we down regulated Clathrin Heavy Chain. As shown in figure 3A, IGF1decreased in Ctr-siRNA cells the amount of IGF-IR in the plasma membrane with a maximum effect at 5 min (mean±SD; 0,73±0,05) followed by a complete replacement to cell surface at 30 min (mean±SD; 0,93±0,05). Cav-1 silenced HaCat cells showed at 5 minutes a lower internalization of IGF-IR that was coupled with a decreased, even if not statistically significant, rate of surface replacement at 30 min compared to cells transfected with a scrambled control siRNA (Fig. 3A). PTRF/Cavin silencing affected significantly IGF-IR plasma membrane recovery at 30 min and reduced IGF-IR internalization at 5 min without reaching a statistical significance (Fig.3A). Clathrin Heavy Chain down regulated cells showed an IGF-IR internalization pattern super imposable to control cells.

**Figure 4:**
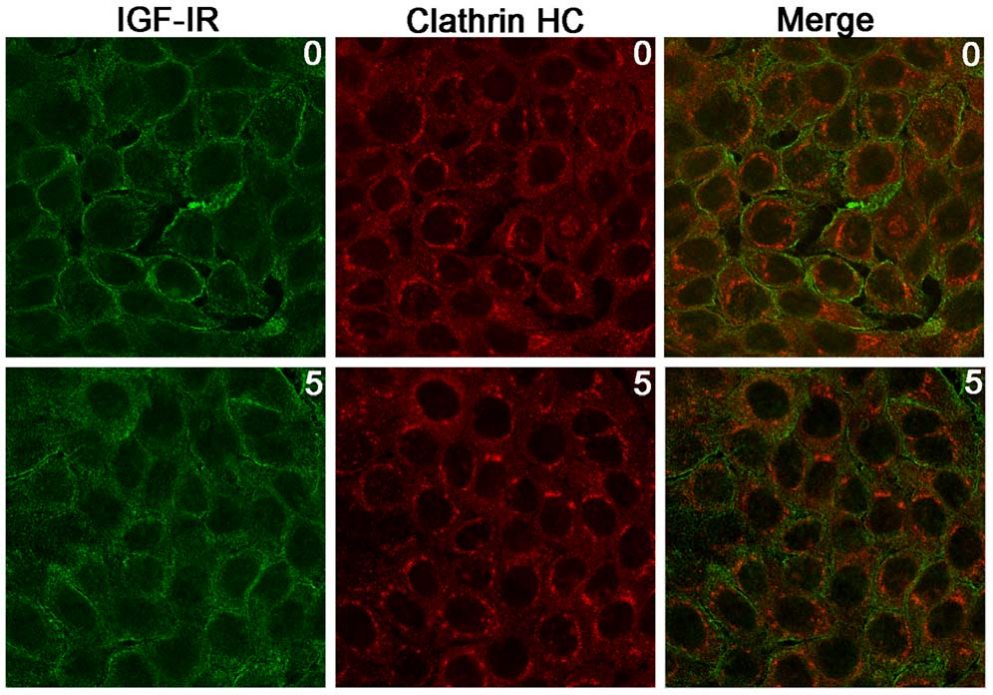
IGF1 does not Induces IGF-IR and Clathrin Heavy Chain co-localization. HaCat cells were treated with IGF1(10 nM) for 5 min, fixed in 4% formaldehyde, permeabilized with Methanol at −20°C, labeled with a rabbit anti-Clathrin Heavy Chain (red) and a mouse anti-IGF-IR (green), antibody and imaged by confocal immunofluorescence microscopy.

To focus on IGF-IR internalization, we used a sensitive biochemical assay based on the use of a reducible biotin reagent. Briefly, Ctr-siRNA, Cav-1 siRNA and PTRF/Cavin siRNA cells were biotinylated at 4°C with NH-SS-biotin and then incubated with IGF1at 37°C for the indicated times to allow internalization. By addition of glutathione (which reduces biotin at the cell surface but does not have access to the internalized proteins) it is possible discriminate between the cell surface and the internalized IGF-IR at different times. Consistently with FACS analysis, biotinylation assay showed that Cav-1 and PTRF/Cavin silencing decreased significantly IGF-IR internalization (Fig. 3B) compared to Ctr and Clathrin HC silenced cells. Cav-1, PTRF/Cavin, and Clathrin HC silencing did not cause any reciprocal significant change in the expression pattern. Also Flotillin-2 and actin expression remained unchanged (fig. 3C). In strong support of the experiments described in figure 3 we did not observe any co-localization between IGF-IR and Clathrin HC protein before and after IGF1 treatment (Fig. 4).

**Figure 5:**
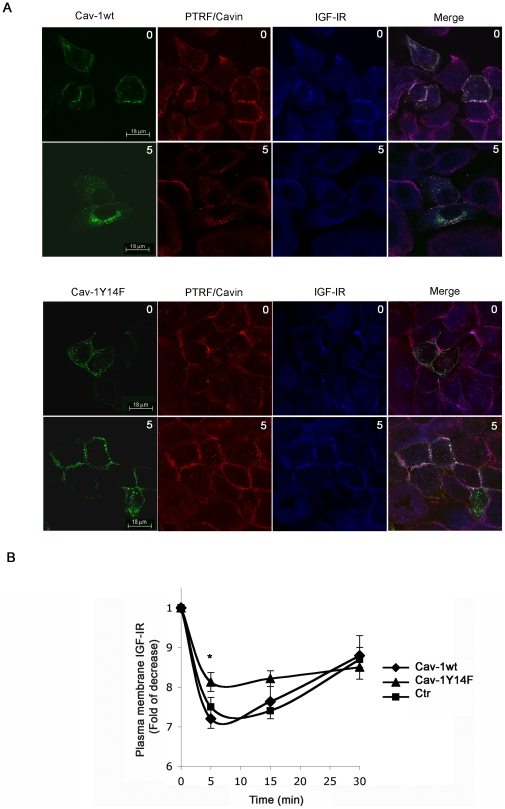
Expression of CavY14F mutant decreases IGF-IR internalization in IGF1 stimulated cells. (A) Hacat cells were transiently transfected with pEGFPN1 Cav-1-wt and pEGFN1 Cav-1Y14F plasmids. 48 hours after transfection serum starved HaCat cells were stimulated with IGF1 10 nM for 5 min, fixed in 4% formaldehyde, permeabilized with Methanol at −20°C, labeled with a rabbit anti-PTRF/Cavin (red) and a mouse anti-IGF-IR (blue) imaged by confocal immunofluorescence microscopy. Merged fields show co-localization (white) of Cav-1wt, PTRF/Cavin, IGF-IR (upper pannels) and Cav-1Y14F, PTRF/Cavin and IGF-IR (bottom panels). (B) 48 hours from the transfection, serum-starved cells were treated with IGF110 nM for the indicated times, trypsined, washed, blocked and incubated with a mouse PE-conjugated IGF-IR.antibody. PE-conjugated IGF-IR labeled cells were analyzed by flow-cytometry to measure plasma membrane IGF-IR expression as described in Materials and Methods. Data are expressed as the mean ± SD. Statistical analysis was performed using Student's *t* test. *p<0.05.

To investigate whether tyrosine phosphorylation of Cav-1 could have a role in IGF-IR internalization we utilized a mutant form of Cav-1 that could not be phosphorylated in tyrosine 14 (Cav1Y14F). Interestingly the mutant-expressing cells showed a decreased ability of IGF-IR to move from the plasma membrane to the intracellular compartment (Fig. 5A). This finding was confirmed also by FACS analysis (Fig. 5B). Hacat Cav1Y14F expressing cells showed a lower rate of IGF-IR internalization and recycling compared to wild type Cav-1 expressing cells and pEGFPN1 transfected cells.

## Materials and Methods

### Cells Culture and transfection

HaCat cells were obtained from cell lines service (Cell line Service-CLS-Germany). HaCat cells were cultured in Dulbecco's modified Eagle's medium supplemented with growth factors, 2 mM glutamine, 2% fetal bovine serum (FBS), 100 U/ml penicillin, and 100 mg/ml streptomycin sulfate at 37°C in 5% CO2. HaCat cells were used between passages 39 and 50, and the medium was changed every 2–3 days. Cav-1 (GenBank accession NM_001753), PTRF/Cavin (GenBank accession NM_012232) and Chlathrin Heavy Chain (GenBank accession NM_004859) silencing was performed using accell siRNA (Dharmacon). CAV-1 target sequences: UGUUGGUCAUUUUAUGUUA, CCCUCAUGCAAAUCAAUUA, GGAUCAACCAUCGCUUUAU, CUUGCAGAAAGAAAUAUAA; PTRF/Cavin target sequences: GCAUGAGAAAUCUAUCUUA,UCAUUGUUUUAAGGGAUUG, CCACGCUCUAUAUUUCGA, GCCUGAUUCUGUUCGGACU; Clathrin-CH target sequences: GAGAAUGGCUGUACGUAAU, UGAGAAAUGUAAUGCG AAU, GCAGAAGAAUCAACGUUAU, CGUAAGAAGGCUCGAGAGU; Accell non targeting-pool (Dharmacon) was used as control. HaCat cells were growth to 60% confluence and transfected the day after with Accell siRNA. After 48 hours, transfection mixtures were replaced by serum free medium O/N and then treated with IGF1(10 nM) for the indicated times.

### Immunoprecipitation and immunoblotting

Serum starved cells were stimulated with IGF110 nM for the indicated time in a CO_2_ incubator at 37°C. Then cells were lysed in Na_2_CO_3_ 0.5 M (pH 11). Immunoprecipitations were carried out as described [25]. Immunoprecipitated proteins were separated on SDS–PAGE and transferred to nitrocellulose. Filters, blocked in 5% dried milk, were incubated with primary antibodies for 2 h at room temperature and washed extensively, and secondary horseradish-peroxidase linked antibodies were added for 1 h at room temperature. Bound antibodies were detected using the enhanced chemiluminescence (ECL) lighting system (Santa Cruz Biotechnology), according to manufacturer's instructions.

### Immunofluorescence

For fluorescence microscopy studies, HaCat cells were plated on glass coverslips harvested for 16 hours and treated with IGF110 nM for the indicated time. Cells were fixed (15 min) with 4% paraformaldehyde, permeabilized with methanol 100% (10 min −20°C), blocked with 3% Bovine Serum Albumin (BSA), 0,1% Triton-X-100 and incubated over night at 4°C with primary antibodies diluted in phosphate-buffered saline (PBS), 0,1% Triton X-100 containing 1% BSA. The following antibodies were used: rabbit anti human IGF-IR (Cell Signaling), or alternatively mouse anti human IGF-IR (clone αIR-3-Oncogene), rabbit anti human PTRF/Cavin (Bethyl), mouse anti Cav-1 (clone 7C8-AbCam). After washing with PBS, the preparations were incubated alternatively with a chicken anti mouse IgG Alexa Fluor 488 antibody, a goat anti rabbit Alexa Fluor 568 or a goat anti mouse Alexa Fluor 633 for 1 hour in PBS containing 1% BSA. Cover slips were mounted using a prolong gold antifade reagent (Invitrogen) and analyzed by confocal microscope. Images were collected using a three-channel TCS SP2 laser-scanning confocal microscope (Leica). Spatial co-localization was analysed by ImageJ 1.34f software (Wayne Rasband, National Institutes of Health, Bethesda, MD, USA).

### Internalization assay: flow cytometry and biochemical analysis

#### Flow cytometry analysis

Serum starved cells were stimulated with IGF110 nM for the indicated time in a CO_2_ incubator at 37°C. Cells were washed twice with phosphate-buffered saline (PBS) and fixed with 4% formaldehyde. 10^6^ cells were blocked in PBS containing 3% BSA for 10 min at 4°C, incubated with a mouse Fluorescein-conjugated anti IGF-IR primary antibody (clone FAB391F-R&D) for 30 minutes at 4° and washed twice with PBS. Additional control samples included cells without antibody and cells incubated with a mouse isotopic control FITC-conjugated IgG antibody (R&D) for 30 minutes at 4°. Cells were pelleted and then resuspended in 0,5 ml of PBS. Flow cytometric analysis was performed on a FC500 flow cytometer (Couter, Hialeah, FL). Fold of decrease of the Mean fluorescence intensity for six independent experiments is shown and error bars represent ± SD.

#### Biochemical analysis

Ctr-siRNA, Cav-1-siRNA, PTRF/Cavin siRNA HaCat cells were serum starved over-night washed with PBS, and processed for internalization assay. The cells cooled on ice and biotinylated with NH-SS-Biotin at 4°C were held at 37°C for indicated times in presence of IGF110 nM. Then residual surface NH-SS biotin was removed by reductive cleavage at 4°C with gluthatione (GSH G4251-1G, from SIGMA)[26]. Biotinylated IGF-IR was immunoprecipitated with IGF-IR antibody and revealed by western blotting using streptavidin-HRP (AbCam) conjugated and ECL.

### Transfection

pEGFPN1-Cav1wt and pEGFPN1-Cav1Y14F were kindly provided by McNiven MA [27] and used to transfect Hacat cells using jetPEI reagent (Polyplus), according to the manufacturer's protocol. 48 hours after transfection serum starved cells were stimulated with IGF1 10 nM for Immunofluorescence and Flow cytometry analysis for the indicated time as described in the corresponding figure legends. FACS analysis of transfected cells was performed with gates set for GFP positive cells.

### Statistical analysis

All experiments were performed at least three times. Statistical differences were assessed by t Student. p<0.05 was considered statistically significant.

## Discussion

Internalization is a mechanism by which RTKs leave the plasma membrane, traveling inside the cell to specific signaling sites. The fine turning of these processes can be altered in cancer cells [28], [29] favouring tumor growth. RTK internalization can follow mainly two pathways: via Clathrin-coated pits and via caveolae [30].

IGF-IR internalization could be Clathrin dependent [5], [6] but some observations have shown a significant role of caveolae in this process [31]. The caveolar mechanisms that regulate internalization and recovery of IGF-IR on plasma membrane remain to be clarified.

Cav-1 and IGF-IR play independent roles in the regulation of cell growth, adhesion and migration but a functional link between these two proteins has been demonstrated [19], [32]. Here we demonstrate that Cav-1 and PTRF/Cavin, a new backbone protein in caveolar structure, regulate IGF-IR internalization and plasma membrane replacement.

In basal condition, we observed that Cav-1 and PTRF/Cavin down regulation did not change the total as well as the surface expression of IGF-IR compared with control cells suggesting that these two proteins are not involved in the post-transductional processes that allow IGF-IR traveling to cell surface.

Cav-1 is a target of IGF-IR and plays a role in IGF-IR signalling pathway [2], [3], [33]. IGF1causes Cav-1 tyrosine phosphorylation and IGF-IR co-localization with Cav-1 in the lipid rafts enriched fractions on plasma membrane [19]. Our previous results showed that Cav-1 silencing impairs the activation of IGF-IR signalling pathway [2]. IGF-IR and Cav-1 co-localize in basal condition and internalize following IGF1stimulation. Co-immunoprecipitation results demonstrate a direct interaction between these proteins with a time course consistent with protein redistribution as shown by immunofluorescence data. These data suggest a role of Cav-1 in the early steps of IGF-IR endocytosis as already shown for IGF1signalling in caveolae [20]. As shown by FACS and biotinylation assay, Cav-1 silencing decreases significantly IGF-IR internalization. This result can be explained by: 1) the fact that the absence of Cav-1 could affect the number as well as the stability of caveolae [34]; 2) a decreased IGF-IR phosphorylation which has been observed in Cav-1 down regulated cells [35].

Silencing of Cav-1 does not abolish completely IGF-IR endocytosis suggesting the presence of alternative pathway. It has been shown that Clathrin coated-pits could participate to IGF-IR internalization [31], [36]. Here the down regulation of Clathrin Heavy Chain did not impair IGF-IR internalization. This conclusion was further supported by the finding that IGF-IR and Clathrin Heavy Chain did not co-localize in Hacat cells in the presence of IGF1.

PTRF/Cavin, a recently identified caveolar protein, could act to maintain caveolae integrity[15], or as transcription terminator [13], as suggested also by the finding that PTRF/Cavin could be recruited to the nucleus by insulin stimulation[17]. Our experiments confirmed that Cav-1 and PTRF/Cavin co-localize in the plasma membrane [15] and show that IGF1increases their association with IGF-IR.

These findings suggest that Cav-1 and PTRF/Cavin could co-operate to determine IGF-IR internalization but with two different roles. In fact we observed two different time courses of association between Cav-1 and PTRF/Cavin with IGF-IR. IGF1binding to IGF-IR also internalizes Cav-1 and PTRF/Cavin with quite different pattern of redistribution of these two proteins as shown by figure 1. These data agree with co-immunoprecipitation of Cav-1 and PTRF/Cavin with IGF-IR.

In Cav-1Y14F overexpressing cells IGF-IR internalization was reduced similarly to silenced Cav-1 cells. This finding extends previous data of Orlichenko et al. [27] that demonstrated a dominant negative effect of Cav-1Y14F mutant on caveolar function and strengthen the importance of Cav-1 tyrosine phosphorylation in RTK compartmentalization.

We demonstrate that both Cav-1 and PTRF/Cavin regulate the surface expression of IGF-IR following IGF1treatment. Since Cav-1 and PTRF/Cavin both regulate caveolae stability, the fact that Cav-1 and PTRF/Cavin reduce IGF-IR endocytosis could be explained by a reduction of caveolae observed in Cav-1 and PTRF/Cavin null cell [15], [34]. This effect could be due also to a structural change as hypothesized by others [15], or to a mislocalization of other caveolar proteins [37].

In our experiments Cav-1 role in IGF-IR recycling remains to be clarified: in fact Cav-1 down regulation consistently slowed the rate of IGF-IR recycling but this effect was not statistically significant. The increase in PTRF-IGF-IR co-immunoprecipitation till 15 min and the effect of PTRF/Cavin silencing on IGF-IR levels suggest that PTRF/Cavin could have a different and specific role compared to Cav-1. We can hypothesize that PTRF/Cavin could play a role during surface IGF-IR recovery and that it could participate to complex mechanisms that regulate recycling. The decrease IGF-IR rate of replacement following PTRF/Cavin silencing in presence of IGF-I, could be related also to increase degradation. Nevertheless we did not observe any change of IGF-IR expression following Cav-1 and PTRF/Cavin silencing during IGF1treatment. Previous studies have demonstrated that down regulation of PTRF/Cavin reduces the stability of Cav-1 [15], [16] and that the absence of Cav-1 causes a decrease expression of PTRF/Cavin [38]. Here we show that Cav-1 and PTRF/Cavin silencing in Hacat cells did not induce any significant reciprocal change in their expression pattern. We can not exclude that later time points after silencing should be required to observe a significant change in the reciprocal expression of these proteins [38]. In conclusion we show for the first time that PTRF/Cavin interacts with IGF-IR and play a role on IGF-IR internalization. Cav-1 and PTRF/Cavin regulate in a distinct manner the balance of surface IGF-IR levels following IGF-I. Then Cav-1 and PTRF/Cavin could represent distinct targets to down regulate IGF-IR action.
